# Effects of Low Temperature Stress on Spikelet-Related Parameters during Anthesis in *Indica–Japonica* Hybrid Rice

**DOI:** 10.3389/fpls.2017.01350

**Published:** 2017-08-08

**Authors:** Yanhua Zeng, Yuping Zhang, Jing Xiang, Norman T. Uphoff, Xiaohua Pan, Defeng Zhu

**Affiliations:** ^1^Key Laboratory of Crop Physiology, Ecology and Genetic Breeding, Jiangxi Agricultural University Nanchang, China; ^2^State Key Laboratory of Rice Biology, China National Rice Research Institute (CAAS) Hangzhou, China; ^3^Cornell Institute for Public Affairs (CIPA), System of Rice Intensification (SRI), Cornell International Institute for Food, Agriculture and Development (CIIFAD), Cornell University, Ithaca NY, United States

**Keywords:** chilling stress, *indica–japonica* hybrid rice, anthesis, pollen germination, spikelet fertility, spikelet flowering

## Abstract

Poor spikelet fertility under low temperature (LT) stress during anthesis limits the possibility of high yield potential in *indica–japonica* hybrid rice, leading to reduced stability of grain yield. However, the cause for it is still unclear. In this study, three *indica–japonica* hybrid rice cultivars, Yongyou9, Yongyou17 (both cold sensitive), and Yongyou538, and one *japonica* inbred rice cultivar, Zhejing88 (cold tolerant), were grown under LT (17°C) and ambient temperature (AT) (25°C) during anthesis to test for their response with respect to spikelet fertility, pollen germination, and spikelet flowering. The results indicated that LT resulted in lower spikelet fertility in cold-sensitive cultivars than in cold-tolerant cultivars. Spikelet fertility was highly correlated with pollen germination on the stigma. The number of pollen grains and germinated pollen were higher in cold-tolerant cultivars than in cold-sensitive cultivars. Pollen fertility and pollen diameter were also higher in cold-tolerant cultivars, although the latter could achieve a high number of spikelets at anthesis in flowering patterns throughout the duration of LT stress. There were significant differences in anther width and volume between genotypes and treatments according to microscopic analyses, but no differences were observed in anther dehiscence. Moreover, variation in the number of pollen grains on stigmas and in spikelet fertility was not related to either the number of spikelets reaching anthesis or anther dehiscence. Overall, improved anther size, better pollen function, and higher spikelet fertility under LT stress were observed in cold-tolerant cultivars than in cold-sensitive cultivars. The results suggest that the increase in spikelet fertility is due to enhanced pollen germination rather than the number of spikelets reaching anthesis.

## Introduction

Rice (*Oryza sativa* L.) is a staple grain crop. Six hundred million tons of rice are produced annually, and rice is widely cultivated throughout the world (IRRI, 2002). Low temperature (LT) is a major environmental factor causing reductions in yield (Boyer, 1982). In general, the mean critical temperature for rice is +4.7°C, and once the ambient temperature (AT) is below 5–10°C, irreversible LT-induced damage to seedling growth and development can occur (Sanchez et al., 2014). During different developmental stages, rice shows growth responses to LT stress. The reproductive stage in rice is a critical period. Farrell et al. (2001, 2006) reported that rice was susceptible to abundant LT-induced damage during the young microspore stage, and 12 h of exposure to LT can cause spikelet sterility. LT-induced spikelet sterility during the microsporogenesis stage is considered genotype dependent, which for cold-tolerant genotypes was a night-time air temperature of 15°C for 4 consecutive days and for cold-sensitive genotypes was a night-time air temperature between 17 and 19°C (Satake, 1969). Hence, developing and verifying cold-tolerant cultivars will be beneficial for overcoming the influence of LT stress on rice growth and development.

Low temperature-induced spikelet sterility, in particular during the reproductive period, could be related to the physiological response mechanisms in the anther, pollen, and stigma. Previous studies have indicated that short anther dehiscence, poor pollen grains, and low pollen germination on stigmas were all highly correlated with spikelet sterility under LT-induced stress (Matsui et al., 2001; Prasad et al., 2006). Moreover, Satake (1976) considered that the main reason for this could be attributed to LT-induced damage of male organs, such as pollen, not of female organs, such as the stigma. Hence, if pollen grains were deficient at anthesis, they could impede pollination, leading to spikelet sterility. These results might be similar to high temperature stress (HTs) in rice. A larger anther and longer stigma (Suzuki, 1981, 1982) were found to contribute to the increase in the basal pore length and found to improve LT tolerance during the reproductive stage, and larger anthers and longer stigmas also ultimately benefit successful pollination (Matsui and Kagata, 2003). In addition, panicle exsertion (PE) (Rang et al., 2011) and peduncle length (PeL) (He et al., 2009) have been reported to be responsible for enhanced spikelet sterility under water stress, as has HTs (Rang et al., 2011), but similar results have not been documented under LT stress.

In most rice genotypes, flowering and anthesis undergo a 5-day period with most spikelets at anthesis between 1000 and 1200 h; the similar patterns of flowering have been reported in *indica* and *japonica* subspecies (Nishiyama and Blanco, 1980; Prasad et al., 2006). Studies have shown that flowering (containing anthesis and fertilization), as a developmental stage in rice, in particular at the booting stage (microsporogenesis), is considered most susceptible to LT (Farrell et al., 2006; Maite and Ellis, 2015). The influences on spikelet flowering in rice have been verified under HTs (Rang et al., 2011). The climatic effects on the timing of day and the duration of flowering in rice also differ (Julia and Dingkuhn, 2012). Jagadish et al. (2007) analyzed the flowering patterns and the time of day of spikelets reaching anthesis under HTs in *indica* and *japonica* rice. Furthermore, spikelet fertility under stress conditions could be generally measured by marking individual spikelets during anthesis, which was adopted as an effective method. *Indica–japonica* hybrid cultivars (Yongyou series) have been successfully bred and have been widely used for rice production due to their exceptionally high grain yield potential. However, they are very susceptible to LT, and can succumb to spikelet sterility. Previous studies have not systematically investigated the effects of LT on spikelet flowering and spikelet fertility in *indica–japonica* hybrid cultivars (Satake, 1976; Nishiyama, 1982; Farrell et al., 2006; Julia and Dingkuhn, 2012; Maite and Ellis, 2015; Zeng et al., 2015).

The overall objectives in the present study were therefore to evaluate spikelet fertility of *indica–japonica* hybrid rice under LT stress and study the main traits of anthesis and pollen related to spikelet sterility. The experiments were conducted under AT (25°C) and LT (17°C) and involved the following: (1) recording the effect of LT on peduncle length (PeL) and PE in rice; (2) studying the influences on spikelet fertility, anther dehiscence, pollen grains, and pollen germination on stigmas under LT during anthesis; and (3) examining the relationship between spikelet flowering and spikelet fertility across cultivars.

## Materials and Methods

### Experimental Design and Crop Husbandry

Pot experiments were conducted during the period from June to November in 2013 and in 2014 at the China National Rice Research Institute (CNRRI), Fuyang district (longitude: 119°55′48′′ E; latitude: 30°2′24′′ N), Zhejiang Province, China. A split-plot design was used for the experiments. Temperature treatments (i.e., LT and AT) were used for the main plots, and cultivars were the subplots with three replications. Half of the plants were established under the AT treatment, and the other half were established under the LT treatment. Each section comprised 40 plants of each cultivar in 20 pots (2 hills per pot, 2 seedlings per hill) under each temperature regime, and each pot was considered a sample. The height and diameter of each rice pot was 33 and 23 cm, respectively. Eight kilograms of paddy soil common to rice planting in the area was placed into each pot. Four rice cultivars were used with different LT sensitivities during anthesis. The growth characteristics of cultivars are described in **Table 1**, including sowing date, growth duration, and flowering dates. The cold-tolerant cultivar Yongyou538, which has a shorter growth duration, was sown 1 week later than the other cultivars to maintain the synchronous time of anthesis.

**Table 1:** Information on four rice cultivars selected for the study.

Cultivar^a^	Ecotype	Gene frequencies		Stress tolerance^b^	Sowing date	Booting^d^	Anthesis	Grain-filling stage
		*Indica*	*Japonica*					
Yongyou9	Subspecies *Indica* ×*Japonica*	0.458	0.542	Chilling sensitive	June 14	September 8–10	September 11–13	October 1–3
Yongyou17	Subspecies *Indica* ×*Japonica*	0.477	0.524		June 14	September 9–10	September 12–13	October 2–3
Yongyou538	Subspecies *Indica* ×*Japonica*	0.459	0.541	Chilling tolerant	June 21^c^	September 1–3	September 4–6	September 24–26
Zhejing88	*O. japonica*	0.100	0.900		June 14	September 2–5	September 5–8	September 25–28

^a^*Cultivars are from the China National Rice Research Institute (CNRRI), China. ^b^The results are derived from those of the study of Zeng et al. (2015). ^c^Yongyou538, which has a shorter growth duration, was sown 1 week later than other cultivars to maintain a synchronous time of flowering. ^d^The booting stage, anthesis, and grain filling were recorded in both 2013 and 2014*.

Pre-germinated seeds were sown in seed trays covered with a matrix containing vermiculite, charcoal, soil, and slow-release fertilizer. After 20 days, the seedlings were transplanted into the pots. The pots were established in an open space under natural environmental conditions except for temperature treatments. Alternate wetting and drying irrigation was conducted throughout the cropping season. Two grams (g) of calcium superphosphate (P_2_O_5_) and 4.0 g of nitrogen, phosphorus, and potassium (NPK) compound fertilizer (15-15-15) were applied to each pot for 1 day before transplanting. One gram of potassium chloride was applied identically at tillering and panicle initiation, and 4.0 g of urea was supplied at planting (50%), at tillering (20%), and at panicle initiation (30%). Pest-, disease-, and weed-related problems were intensively controlled. Other management practices for high grain yield cultivation were in accordance with local recommendations. At the end of productive tiller stage, redundant tillers were removed, leaving approximately 12 large tillers.

### Stress Treatments

**FIGURE 1:**
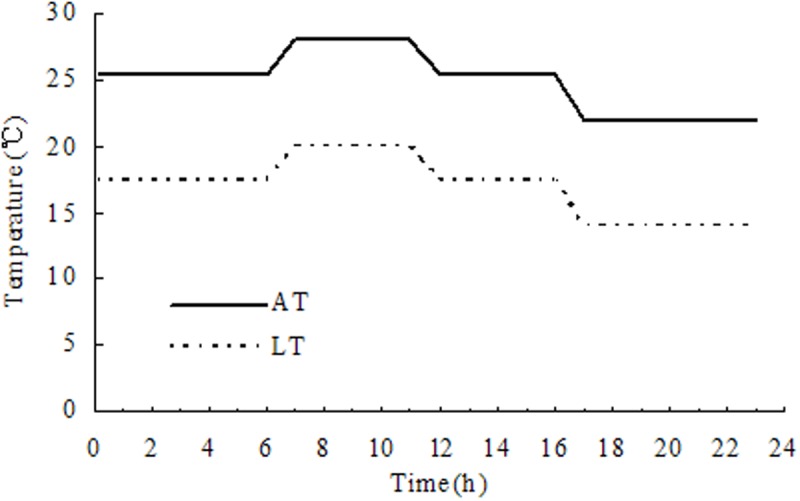
Temperature treatments, AT, ambient temperature; LT, low temperature.

On the day of spikelet flowering (i.e., anthers appearing or spikelets expanding), the plants of each cultivar were transferred to growth chambers (PGV-36, Conviron Corporation, Winnipeg, MB, Canada) with automatically controlled temperatures using sensing elements. The temperature threshold of the rice plants during the reproductive growth stage (booting stage and heading) is often described as 17°C (Satake, 1969; Farrell et al., 2006). Therefore, in this study, LT was set at 17°C, and the growth chamber was held at an average LT of 17°C (20/14°C max/min; actual: 16.7°C [*SD* = 1.16]). The plants of the AT treatment were maintained at an elevated average temperature of 25°C (28/22°C max/min; actual: 23.7°C [*SD* = 0.64]) for five consecutive flowering days, such that the AT was nearly equal to the natural environmental temperature during the past 10 years during flowering days, as described by Zeng et al. (2015). The time window of the temperature treatment day was divided into four time frames on five consecutive flowering days (**Figure 1**), and the relative humidity (RH) was 75% (actual: 81.7% [*SD* = 1.10]). Plants were spaced at a distance of approximately 15–20 cm (0.15–0.20 m) to reduce crowding and shading effects. Five days after the temperature treatment, the plants were placed under natural environmental conditions. Temperature and RH were both monitored independently through recordings by a micrometeorological station (HOBO, H08-002-21, Onset Computer Corporation, Barnstable, MA, United States) with different sensors/loggers every 10 s and averaged for each 30 min. Temperatures surrounding the plants were controlled and maintained consistently within the set points of 0.5°C for temperature and 5% for RH in order to reduce any chamber effects on the observations recorded of plants. In addition, the level of photosynthetic photon flux density for each temperature treatment was maintained at 640 μmol m^-2^ s^-1^. Natural environmental conditions were adopted for plant growth and development, and no unusual HT or LT was observed.

### Measurements and Data Collection

In the study, five pots (10 plants) were used for testing PeL, PE, and wrap-around panicle length (WPL) under both LT and AT treatments; another five pots were used to determine spikelet fertility and spikelet flowering, while the remaining 10 pots in the set were used for the collection of spikelets for microscopic analyses.

#### PeL, PE, and WPL

PeL and PE were determined using a ruler according to the methods of Rang et al. (2011). The elongation of panicle exsertion (EPE) was calculated as the ratio of PeL to the duration of exposure to LT. EPE was to express the degree of LT stress on peduncle exsertion. PeL, panicle length (PaL), and WPL were measured in 2013.

#### Microscopic Analysis of Spikelets

Eight to 10 uniform panicles of each pot were marked for exposure to temperature treatment during anthesis. Twenty to 25 spikelets, positioned on the three primary branches on the top rachis in each panicle (Matsui and Kagata, 2003), were collected simultaneously between 1030 and 1200 h and between 1300 and 1400 h for different treatments and cultivars every day. Therefore, spikelets that approximately opened (i.e., with an observable anther) on the main tiller and on some primary tillers were both marked using black acrylic paint. Then, marked spikelets were collected in brown glass vials filled with formalin–acetic acid–alcohol (FAA) fixative (including 90 mL of 70% ethanol, 5 mL of acetic acid, and 38% formaldehyde) after flowering and pollination, following the methods of Jagadish et al. (2010). The spikelets collected from the tillers were used for microscopic analyses (i.e., anther dehiscence, total pollen grain number on stigmas, and pollen germination), excluding spikelet fertility estimations.

The spikelets were dissected under a stereoscopic microscope (Olympus SZX7, Olympus Corporation, Tokyo, Japan). Then, using a digital camera (DP70) affixed to an Axioplane 2 microscope (Carl Zeiss, Oberkochen, Germany) at 100× (**Figure 5**), images were taken following the methods of Jagadish et al. (2010). The anthers were isolated from the fixed spikelets to score the numbers of dehisced anthers, which were described as full anther dehiscence (FAD) if both apical and basal pores were present, partial anther dehiscence (PAD) if only one pore was present, and anther indehiscence (AID) if no pore was present. The ratio of the number of dehiscent anthers to the total number of anthers (containing both dehiscent and indehiscent anthers) was calculated and described as anther dehiscence (Rang et al., 2011). Anther length and width were also determined, and anther volume was calculated using the following equation: *V* = 0.34 *LW*^2^ (Nishiyama, 1982). Anthers were separated on a glass slide and colored with 1% I_2_–KI with the aid of a stereoscopic microscope (Olympus SZX7, Olympus Corporation, Tokyo, Japan), and the number of pollen grains and pollen diameter were recorded. Stigmas separated from spikelets with minimum disturbance were cleared using 8 N NaOH for 3–5 h at room temperature, after which they were stained with 0.05% aniline blue dissolved in a solution of 0.1 M dipotassium phosphate for 5–10 min. The pollen grains and germination on the stigma were recorded using a fluorescence microscope (Olympus BX43, Olympus Corporation, Tokyo, Japan). The amount of germinated pollen on the stigma was scored according to the procedures of Jagadish et al. (2010), and the per cent pollen germination (PPG) was calculated as the percentage of germinated pollen of the total amount of pollen (germinated + non-germinated). All the above measurements were performed using calibrated Image Pro-Plus 6.0 software (Media Cybernetics, Inc., Bethesda, MD, United States).

#### Spikelet Fertility and Spikelet Flowering

The main-tiller panicles on the day before the heading stage were selected and tagged for each cultivar in the treatments, and then the panicles were harvested at physiological maturity. PaL and the number of filled (i.e., completely or partially filled with grain) and unfilled grains per panicle were both determined, and spikelet fertility (seed set) was recorded for 2 years following the methods of Mohammed and Tarpley (2009). Each grain was evaluated for whether it was filled or not by pressing the grain between the forefinger and the thumb. Since the grain filling of the spikelets on different positions of the panicle was not synchronous, spikelet fertility ultimately differed between the upper part and the lower part. Panicles were separated into the upper (i.e., superior grains), middle, and lower (i.e., inferior grains) parts described as spikelet positions in accordance with the protocol of Yang et al. (2006) to test the effects on spikelet fertility in each part. Spikelet fertility was evaluated by dividing the number of filled grains by the total number of grains (i.e., florets) and was described as the per cent. In addition, in 2014, the effect of temperature on spikelet opening, marked by different tinctorial acrylic paints, was also examined on five consecutive flowering days in accordance with the methods of Jagadish et al. (2007, 2008). The effect of flowering patterns was also identified using blue paint to distinguish spikelets undergoing anthesis at different time periods between 0830 and 1630 h Beijing Summer time (BST), and spikelets were counted every 2 h on the third flowering day. Spikelet flowering in this study was described as the per cent of the numbers of spikelets at anthesis of the total number of spikelets in the panicle (anthesis + non-anthesis) per 2 h period on the third day of anthesis in the four cultivars among the treatments. Spikelet fertility of the marked panicles was also ultimately recorded.

### Statistical Analysis

All data of several traits were analyzed as a spilt-plot design, in which the main plot consisted of temperature treatments (low temperature and ambient temperature) with three replications and the subplots consisted of cultivars, using PROC Generalized Linear Mixed Models in the SAS 9.1 (Cary, NC, United States). The data regarding morphology at anthesis, anther dehiscence, pollen germination traits, spikelet flowering and spikelet fertility were used to test the analysis of variance (ANOVA) of temperature treatments, cultivars and their interaction. Linear regressions among pollen traits, anther dehiscence, spikelet fertility, and spikelet flowering were also conducted using GLM procedures. The values in this study were expressed as the means and errors. The means were examined by Tukey’s least significant difference (LSD) test to compare the differences at the probability level of 0.05.

## Results

### PeL, PE, and WPL

Elongation of panicle exsertion significantly decreased (*P* < 0.05) among cultivars subjected to LT stress compared with AT (**Table 2**). The interaction between cultivars and treatments was also significantly different. Across treatments, the EPE of cold-sensitive cultivars, Yongyou9 and Yongyou17, was higher than that of cold-tolerant cultivars, Yongyou538 and Zhejing88. Similarly, the PE of all cultivars also significantly (*P* < 0.05) decreased by 44.3% under LT (**Table 2**); Yongyou9 had the highest PE, ranging from 32.0 to 58.0%. PeL among the cultivars was consistently maintained in response to LT stress and was 68.1% longer in cold-sensitive cultivars than in cold-tolerant cultivars. Across all the cultivars, PeL was reduced by 6.81 cm under LT, compared with AT. Furthermore, the WPL of Yongyou17 was significantly longer by 114.1% under LT than under AT, but there was no difference (*P* > 0.05) in Yongyou9. Compared with AT, the percent of spikelets trapped inside panicle was enhanced by 3.65% in all the cultivars under LT.

**Table 2:** Effects of LT and AT on morphological traits of the panicles in four cultivars.

Trait	Treatments	Yongyou9	Yongyou17	Yongyou538	Zhejing88	ANOVA		
						*G*	*T*	*G* ×*T*
Elongation of panicle exsertion (cm⋅d^-1^)	LT	3.39 ± 0.18^b^	2.65 ± 0.22^b^	1.60 ± 0.04^b^	1.72 ± 0.12^b^	^∗∗^	^∗∗^	^∗∗^
	AT	5.61 ± 0.28^a^	5.04 ± 0.30^a^	3.70 ± 0.17^a^	3.11 ± 0.15^a^			
Wrap-around panicle Length (cm)	LT	1.13 ± 0.32^a^	3.79 ± 0.26^a^	2.69 ± 0.36^a^	1.47 ± 0.59^a^	ns	ns	ns
	AT	1.99 ± 0.44^a^	1.77 ± 0.33^b^	3.10 ± 0.36^a^	1.90 ± 0.25^a^			
Peduncle length (cm)	LT	16.58 ± 0.97^a^	9.95 ± 0.94^b^	7.67 ± 0.38^b^	8.11 ± 0.57^b^	ns	^∗^	ns
	AT	17.39 ± 0.93^a^	18.38 ± 1.01^a^	18.49 ± 0.83^a^	15.30 ± 0.63^a^			
Panicle exsertion (%)	LT	68.59 ± 3.97^b^	43.41 ± 3.69^b^	35.37 ± 2.36^b^	51.98 ± 2.21^b^	ns	^∗∗^	ns
	AT	89.47 ± 1.83^a^	95.65 ± 2.64^a^	87.54 ± 1.82^a^	85.16 ± 2.89^a^			

*Data indicate the means ± standard errors, *n* = 60. Means within a column followed by a different lowercase letter differ significantly at a level of 0.05. G: genotypes; T: treatments. ^∗^*P* < 0.05, ^∗∗^*P* < 0.01, ns, not significant*.

### Anther Dehiscence and Anther Characteristics

**FIGURE 2:**
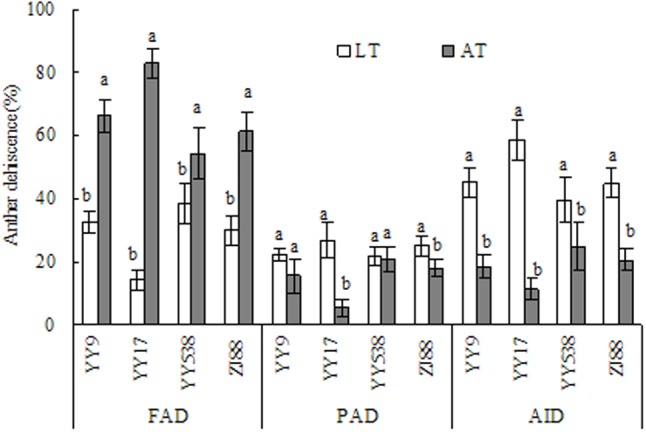
Effects of LT and AT on anther dehiscence of rice plants (*n* > 60 anthers). The three parameters were analyzed separately for each anther dehiscence. Bars with different lowercase letters denote a particular anther dehiscence across cultivars, which differ at a significance level of 0.05. Bars indicate standard error. FAD, full anther dehiscence; PAD, partial anther dehiscence; AID, anther indehiscence; YY9, Yongyou9; YY17, Yongyou17; YY538, Yongyou538; ZJ88, Zhejing88; AT, ambient temperature; LT, low temperature.

Low temperature have an influence on anther dehiscence (including FAD, PAD, and AID), and there were obvious differences among cultivars (**Figure 2**). All cultivars showed less FAD under LT than under AT, and a significant difference (*P* < 0.05) was observed in the majority of the cultivars. However, the cultivars had higher PAD under LT than under AT, and Yongyou17 was significantly affected (*P* < 0.05). Overall, LT increased anther indehiscence in cold-sensitive cultivars.

Anther size (anther length, width, and volume) varied among temperature treatments and cultivars (**Table 3**), and the effects on the cultivars were significantly different (*P* < 0.05). Anther width and volume indicated the same decreasing trends under LT compared with those under AT; however, a significant difference was observed in cold-sensitive cultivars regarding anther volume. There were no significant differences among cold-tolerant cultivars.

**Table 3:** Effects of LT on the anther size of rice plants (*n* = 60) during the flowering period.

Trait	Treatments	Yongyou9	Yongyou17	Yongyou538	Zhejing88	ANOVA		
						*G*	*T*	*G* ×*T*
Anther length (mm)	LT	2.44 ± 0.03^a^	2.28 ± 0.04^a^	2.11 ± 0.04^a^	2.00 ± 0.07^a^	^∗∗^	ns	ns
	AT	2.47 ± 0.03^a^	2.30 ± 0.01^a^	2.04 ± 0.01^a^	2.01 ± 0.04^a^			
Anther width (mm)	LT	0.64 ± 0.03^b^	0.58 ± 0.04^b^	0.67 ± 0.04^a^	0.57 ± 0.04^a^	^∗∗^	ns	ns
	AT	0.70 ± 0.02^a^	0.63 ± 0.02^a^	0.70 ± 0.02^a^	0.62 ± 0.02^a^			
Anther volume (mm^3^)	LT	0.34 ± 0.04^b^	0.26 ± 0.04^b^	0.32 ± 0.04^a^	0.22 ± 0.04^a^	^∗∗^	ns	ns
	AT	0.41 ± 0.03^a^	0.31 ± 0.02^a^	0.34 ± 0.02^a^	0.26 ± 0.02^a^			

*Data indicate the means ± standard errors. Means within a column followed by a different lowercase letter differ significantly at a level of 0.05 for a particular parameter. G: genotypes; T: treatments. ^∗^*P* < 0.05, ^∗∗^*P* < 0.01, ns, not significant*.

### Spikelet Fertility and Pollen Grain on the Stigma

There were significant (*P* < 0.01) differences among cultivars and temperature treatments on spikelet positions regarding spikelet fertility (**Table 4**). Spikelet fertility significantly (*P* < 0.05) decreased in Yongyou9 (9.5%) and Yongyou17 (12.5%) under LT (**Table 4**), whereas no significant differences were observed in Yongyou538 and Zhejing88. Based on spikelet position, LT also significantly reduced spikelet fertility in the upper, middle, and lower parts of the panicle, with a significant impact on cold-sensitive cultivars, particularly in the upper part of the panicle.

**Table 4:** Effects of LT and spikelet position on the spikelet fertility of rice plants (%).

Spikelet position	Treatments	Yongyou9	Yongyou17	Yongyou538	Zhejing88	ANOVA		
						*G*	*T*	*G* ×*T*
Whole	LT	84.4 ± 1.4^b^	83.3 ± 1.4^b^	89.1 ± 0.3^a^	95.5 ± 0.7^a^	^∗∗^	^∗∗^	^∗∗^
	AT	93.2 ± 0.9^a^	95.2 ± 0.6^a^	92.5 ± 1.4^a^	96.5 ± 0.6^a^			
Upper	LT	83.3 ± 2.0^b^	77.7 ± 1.9^b^	91.9 ± 0.5^a^	96.2 ± 0.9^a^	^∗∗^	^∗∗^	ns
	AT	96.1 ± 0.8^a^	96.3 ± 0.4^a^	95.7 ± 0.8^a^	98.0 ± 0.3^a^			
Middle	LT	84.3 ± 1.8^b^	85.5 ± 1.9^b^	86.7 ± 1.7^b^	96.0 ± 0.7^a^	^∗∗^	^∗∗^	^∗^
	AT	92.9 ± 0.9^a^	95.2 ± 0.7^a^	91.9 ± 1.6^a^	96.5 ± 0.6^a^			
Lower	LT	88.8 ± 1.2^b^	86.8 ± 1.2^b^	81.9 ± 2.7^b^	94.7 ± 1.2^a^	^∗∗^	^∗∗^	^∗∗^
	AT	91.6 ± 1.4^a^	94.0 ± 1.2^a^	90.6 ± 1.9^a^	95.7 ± 1.2^a^			

*Data indicate the means ± standard errors, *n* = 25. Means within a column followed by a different lowercase letter differ significantly at a level of 0.05. G: genotypes; T: treatments. ^∗^*P* < 0.05, ^∗∗^*P* < 0.01, ns, not significant*.

**FIGURE 3:**
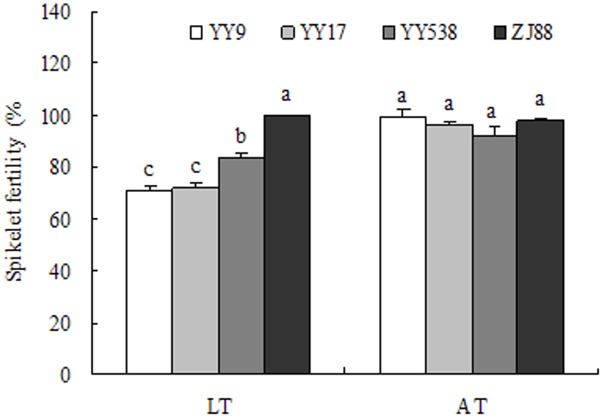
Spikelet fertility during five consecutive days of anthesis of four rice varieties under LT and AT. On different days, individual spikelet fertility was marked with different colored acrylic paints (*n* = 10), and the fertility of spikelets undergoing anthesis was scored separately. Bars with different lowercase letters denote spikelet fertility, which differ at a significance level of 0.05. Smaller bars indicate standard error. AT, ambient temperature; LT, low temperature.

Spikelet fertility on five consecutive flowering days decreased significantly due to being subjected to LT for both Yongyou9 and Yongyou17. Zhejing88 and Yongyou538, however, exhibited much higher spikelet fertility under LT than did Yongyou9 and Yongyou17 (**Figure 3**). Spikelet fertility under AT was not significantly different among cultivars. There was less spikelet fertility reduction in Yongyou9 (-29.4%) and Yongyou17 (-25.5%) under LT than in Yongyou538 (-9.8%) and Zhejing88 (2.1%).

**FIGURE 4:**
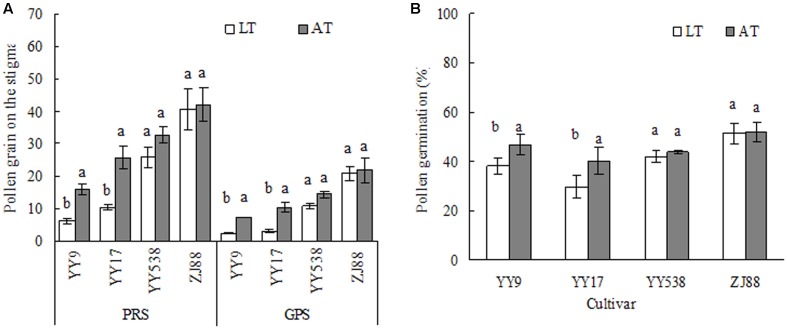
Effects of LT and AT on the number of pollen grains on stigmas (**A**, *n* > 60 spikelets) and per cent pollen germination **(B)** of four rice cultivars. Smaller bars with different lowercase letters indicate significant differences at the level of 0.05. Smaller bars indicate standard error. PRS, pollen reception on the stigma; GPS, number of germinated pollen grains on stigmas; AT, ambient temperature; LT, low temperature.

**FIGURE 5:**
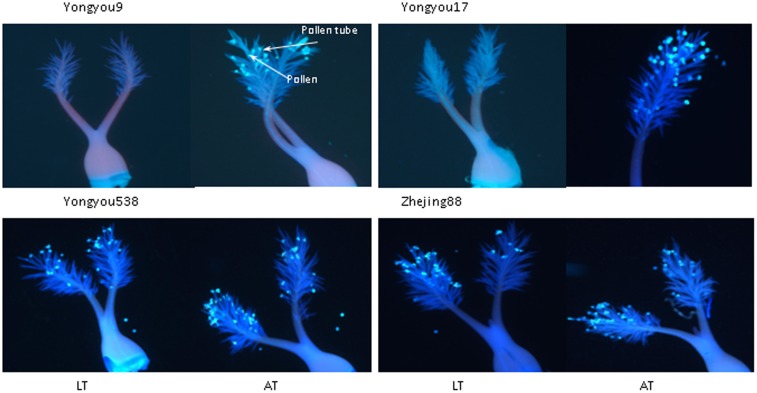
Pictorial illustration of LT and AT affecting the number of pollen grains and pollen germination of four rice genotypes. The luminous circle symbol indicates pollen, and the threadlike light line represents the pollen tube. Pollen was recorded as germinated when the length of pollen tube was at least equivalent to the pollen diameter. AT, ambient temperature; LT, low temperature.

Variational differences in the numbers of pollen grains, germinated pollen grains, and PPG between treatments followed the same trends in all cultivars (**Figure 4**). These traits significantly decreased under LT in Yongyou9 and Yongyou17 compared with those under AT but not in Yongyou538 and Zhejing88. The results are shown as pictorial illustrations of pollen count and pollen germination (**Figure 5**). There were significant differences between the cultivars and treatments and their interaction for pollen fertility and diameter (**Table 5**). Moreover, under LT stress, pollen fertility and diameter significantly decreased in cold-sensitive cultivars compared with those under AT, but no differences in cold-tolerant cultivars were observed (*P* > 0.05). Pollen fertility and diameter were both positively related to the number of germinated pollen grains (*r* = 0.77 and *r* = 0.79, respectively; *n* = 15).

**Table 5:** Effects of LT on pollen fertility and pollen diameter (*n* = 60) during the flowering period.

Trait	Treatments	Yongyou9	Yongyou17	Yongyou538	Zhejing88	ANOVA		
						*G*	*T*	*G* ×*T*
Pollen fertility (%)	LT	74.96 ± 1.29^a,b^	72.26 ± 3.63^b^	85.13 ± 1.22^a^	87.13 ± 0.63^a^	^∗∗^	^∗∗^	^∗^
	AT	86.01 ± 1.11^a^	80.94 ± 2.72^a^	87.32 ± 1.55^a^	88.88 ± 0.95^a^			
Pollen diameter (μm)	LT	42.57 ± 0.46^b^	41.40 ± 0.10^b^	44.26 ± 0.52^a^	44.66 ± 0.21^a^	^∗∗^	^∗∗^	^∗^
	AT	44.07 ± 0.43^a^	43.90 ± 0.59^a^	44.90 ± 0.28^a^	44.94 ± 0.31^a^			

*Data indicate the means ± standard errors. Means within a column followed by a different lowercase letter differ significantly at a level of 0.05. G: genotypes; T: treatments. ^∗^*P* < 0.05, ^∗∗^*P* < 0.01, ns, not significant*.

### Spikelet Flowering at Anthesis

**FIGURE 6:**
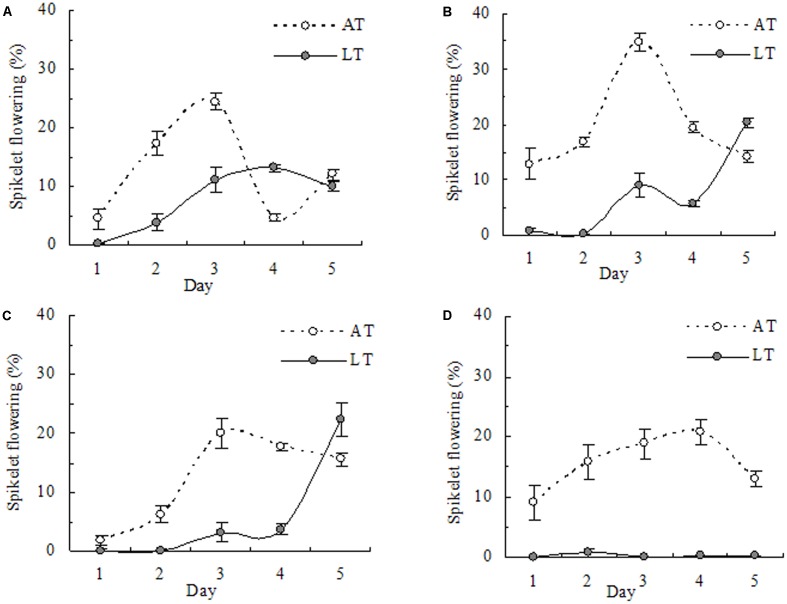
Spikelet flowering per day during five consecutive days of anthesis in *indica–japonica* hybrid rice Yongyou9 **(A)**, Yongyou17 **(B)** and Yongyou538 **(C)** and in *japonica* rice Zhejing88 **(D)** under LT and AT. Individual spikelet flowering per panicle was marked with different colored acrylic paints on different days (*n* = 10), and spikelet fertility was scored separately. Smaller bars indicate standard error. AT, ambient temperature; LT, low temperature.

The time of day of spikelets undergoing anthesis for 5 days under AT showed similar trends among cultivars, and flowering peaked on the third (or fourth) day (**Figure 6**). On average, LT significantly reduced the total number of spikelets at anthesis in all cultivars. However, spikelet flowering under LT was higher in Yongyou9 and Yongyou17 than in Yongyou538 and almost decreased to 0% during the 5-day flowering period for Zhejing88.

**FIGURE 7:**
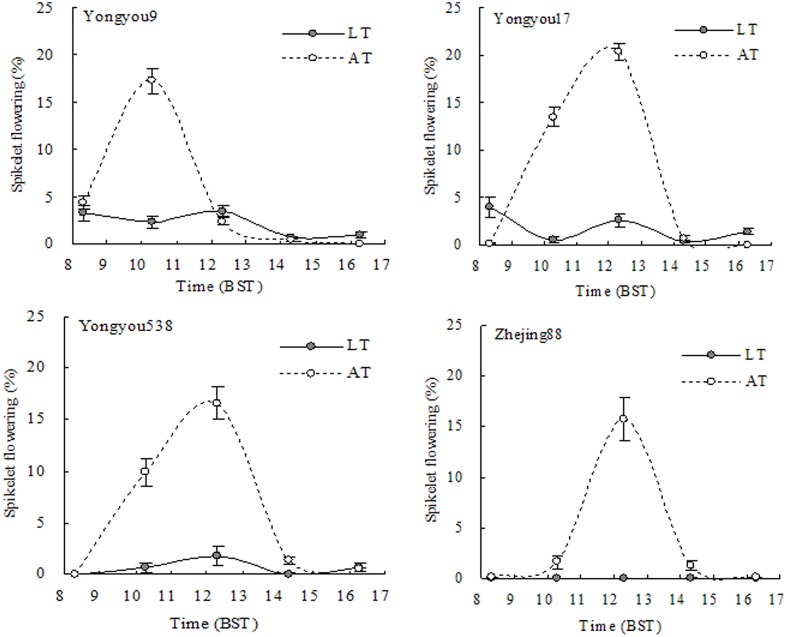
Spikelet flowering every 2 h on the third day of anthesis in four rice genotypes under LT and AT. Individual spikelets flowering per panicle at different times of day during anthesis were marked with different colored acrylic paints (*n* = 10), and spikelet fertility was scored separately. Smaller bars indicate standard error. BST, Beijing Summer time; AT, ambient temperature; LT, low temperature.

The pattern of flowering under AT showed similar trends among cultivars of increasing but then decreasing on the third flowering day, peaking at 1230 or 1030 h (Yongyou9) (**Figure 7**). However, LT significantly reduced spikelet flowering between 1030 and 1230 h. Under LT, spikelet flowering decreased on the third flowering day and was higher on average in Yongyou9 (2.14%) and Yongyou17 (1.82%) compared to that of Yongyou538 (0.63%) and Zhejing88 (0.01%).

Spikelet fertility was positively correlated with the number of pollen grains, germinated pollen grains, and PPG but not with anther dehiscence or spikelet flowering (**Table 6**). Moreover, anther dehiscence was not correlated with spikelet flowering (*P* > 0.05).

**Table 6:** Relationship among pollen count on the stigma, pollen germination, anther dehiscence, spikelet fertility, and spikelet flowering (*n* = 15).

Trait	Anther dehiscence (%)	Spikelet fertility (%)	Spikelet flowering (%)
Number of pollen grains on the stigma	-0.081^ns^	0.679^∗∗^	-0.372^ns^
Number of germinated pollen grains on the stigma	0.042^ns^	0.779^∗∗^	-0.344^ns^
Pollen germination (%)	0.410^ns^	0.759^∗∗^	-0.116^ns^
Anther dehiscence (%)	—	0.311^ns^	—
Spikelet flowering (%)	0.350^ns^	-0.390^ns^	—

^∗^*P < 0.05, ^∗∗^*P* < 0.01, ns, not significant*.

## Discussion

With changing global climate, extreme cold conditions will be more frequent in the future, which make rice subject to adverse abiotic stresses. Improving the temperature stress tolerance in rice at anthesis during the susceptible reproductive stage is beneficial for adapting to highly dynamic climatic conditions in the future (Horie et al., 1996). In addition, increasing the absolute stress tolerance of rice could facilitate important physiological processes (e.g., anther dehiscence, pollen germination, pollination, and fertilization) being carried out with high spikelet fertility under stress (Jagadish et al., 2008, 2010; Rang et al., 2011). Rang et al. (2011) considered that spikelet fertility might be largely determined by morphological conditions at anthesis under different abiotic stresses. PE, which was primarily affected by PeL, could be described as affecting rice spikelet fertility when exposed to LT. However, in the present study, the panicles were partially exserted under LT; some spikelets within the panicles were still stuck in the leaf sheath of the four cultivars regardless of the degree of cold tolerance and EPE (**Table 2**). Interestingly, we found that PeL decreased significantly (*P* < 0.05) under LT in all cultivars apart from Yongyou9. These results were similar to the previous findings under HTs (Rang et al., 2011).

Although spikelets stuck in the leaf sheath could be reflective of spikelet fertility through altered anthesis and fertilization (O’Toole and Namuco, 1983; Cruz and O’Toole, 1984), the physiological process of spikelet fertility under LT did not invariably coincide with phenology. In general, anther dehiscence can affect the number of pollen grains on the stigma (Rang et al., 2011). In the present study, anther indehiscence under LT was higher in cold-sensitive cultivars than in cold-sensitive cultivars (**Figure 2**). It was mainly due to a sharply reduced FAD, resulting in decreased pollen grains on the stigma. Liu et al. (2006) reported that the rupturing of the septa, the expansion of locule walls, the swelling of pollen grains, and then the rupturing of the stomium were all highly related to anther dehiscence. However, in the present study, this phenomenon was not observed (**Table 6**). The reason was that anthers still dehisced under stress due to spikelet flowering; pollen grains could not swell, and they lost viability and did not shed, resulting in unfertilized pollen (Farrell et al., 2006; Liu et al., 2006; Jagadish et al., 2010). There was no relationship between greater anther dehiscence and more pollen grains on the stigma, as was described by Jagadish et al. (2010). However, spikelet fertility under cool temperature conditions was strongly correlated with large anthers (Suzuki, 1981), and anther length, width and area benefit increasingly large pollen grains with respect to fertilization (Farrell et al., 2006). Our findings also showed that anther volume, which was significantly affected in cold-sensitive cultivars, was more closely related to anther width (*r* = 0.96, *P* < 0.01; *n* = 17, data not shown) than anther length (*r* = 0.69, *P* < 0.01) under LT. In addition, spikelets scored for high fertility had at least 10 or more than 20 germinated pollen grains, which could be identified as the threshold number (Jagadish et al., 2010). It was therefore thought that cold-tolerant cultivars obtained more germinated pollen on the stigma (**Figure 4A**), and increased seed set compared with that of cold-sensitive cultivars under LT was ultimately confirmed (**Table 4**). It has been reported that pollen germination alters spikelet fertility, ultimately affecting rice productivity under high night-time temperatures (Mohammed and Tarpley, 2009) as well as under LT. Liu et al. (2006) reported that improved pollen stickiness, which could restrict pollen grains from shedding whether the anther pores dehisced or not, might affect pollen count on the stigma. Our results suggest that a small pollen count and germinated pollen grains under LT were observed in cold-sensitive cultivars (**Figures 4**, **5**), mainly due to poor pollen fertility and shortened pollen diameters (**Table 5**). Moreover, there was strong positive relationship between spikelet fertility and pollen germination (**Table 6**). However, the mechanism controlling high pollen germination with low anther dehiscence in spikelets under LT stress is not clear.

Although a uniform level of PE, PeL, and WPL between treatments was observed among the cultivars, rice plants exposed to LT did have lower spikelet fertility across cold-sensitive cultivars (**Table 4**). This was caused by different spikelet positions within the panicle (*P* < 0.05); the upper parts of the panicle under LT stress and LT alone could especially aggravate poor spikelet fertility. However, under stress conditions, the grains at the upper parts of the panicle are usually filled primarily (Stansel, 1975). The upper parts are also exposed to LT first, which can enhance the extent of cold-induced damage and lead to spikelet sterility, even when the panicles are exserted. However, the lower and middle parts of the panicle were correspondingly subjected to less damage due to the site of PE. Similar results on the grain characteristics of rice under high night-time temperatures and spikelet position can be obtained from the report of Mohammed and Tarpley (2010). Furthermore, the effect of spikelet position within the panicle on spikelet fertility across cultivars was also related to the exsertion of the panicle.

The cumulative negative influence of extended LT stress on consecutive flowering days was examined in terms of spikelet fertility (seed set) using the individual spikelets marked. In the study, we observed a significant reduction in spikelet fertility with the duration of flowering in cold-sensitive cultivars but not in cold-tolerant cultivars (**Figure 3**). This result was in accordance with that in the studies of Jagadish et al. (2007) and Rang et al. (2011) on the effects of HTs. Flowering under AT peaked within a 5-day period earlier in *indica–japonica* hybrid cultivars than in *japonica* rice (**Figure 6**). The former cultivars contain the *indica* gene besides the *japonica* gene (Prasad et al., 2006), resulting in early flowering, even though the pattern of flowering, which reached peaked between 1030 and 1230 h, on the third day was similar among cultivars (**Figure 7**). Compared with cold-sensitive cultivars, cold-tolerant cultivars still had fewer numbers of flowering spikelets under LT (**Figures 6**, **7**), allowing floral organs to avoid damage from LT. Jagadish et al. (2007) proposed that more spikelet anthesis is correlated with a higher spikelet fertility under HTs. However, this phenomenon was not verified in our study, because spikelet flowering did not significantly affect spikelet fertility (**Table 6**). Hence, our results stated that spikelet fertility was not the consequence of spikelet flowering. It had been understood that the normal action of dehisced pollen sacs in anthers and pollen vitality might be affected by LT stress as well as by HTs a day ahead flowering (Matsui and Omasa, 2002). It is possible that in cold-susceptible cultivars such as Yongyou9 and Yongyou17, other processes that occur before fertilization were affected, such as pollen shrinkage and anther volume reduction (Farrell et al., 2006) as well as effects on pollen tube growth rate and pollen germination (Kakani et al., 2002; Coast et al., 2015). This could confirm that high pollen germination was observed in Yongyou538 and Zhejing88. Nonetheless, it is unlikely that ovary development was unaffected after fertilization, although spikelets subjected to LT after opening were fertile in both cold-sensitive cultivars and cold-tolerant cultivars. This phenomenon could also result in spikelet sterility.

In general, *indica* species are more tolerant to HTs; conversely, *japonica* species are more tolerant to LT stress (Farrell et al., 2006). Moreover, *indica–japonica* hybrid rice, which simultaneously contains *indica* and *japonica* genes, was sensitive to temperature fluctuations, resulting in spikelet sterility under LT, despite the neutral alleles at the two loci being used to solve pollen sterility in *indica–japonica* hybrid rice (Lu et al., 2000). In this study, *indica–japonica* hybrid rice cultivars, which were tested for the gene frequencies of *indica* and *japonica* using SSR molecular markers (data not shown), indeed had different responses to LT. Our study may provide evidence of *indica–japonica* hybrid rice adapting to extreme climatic changes for breeding purposes.

## Conclusion

Our study has shown that LT induces spikelet sterility by shrinking anther size and decreasing pollen function (the number of pollen grains, pollen fertility, and pollen germination on stigmas) in cold-sensitive cultivars during anthesis. We found that spikelet fertility was strongly correlated with pollen germination rather than spikelet flowering across cultivars and treatments. Spikelet fertility was not attributed to the patterns of flowering and the number of spikelets reaching anthesis. Therefore, this aspect should be considered for breeding *indica–japonica* hybrid rice varieties to tolerance during flowering.

## Author Contributions

DZ conceived and designed the experiments. YPZ, JX, NU, and XP provided experimental opinions and assistance. YPZ and YHZ performed the experiments. YHZ analyzed the data and wrote the paper.

## Conflict of Interest Statement

The authors declare that the research was conducted in the absence of any commercial or financial relationships that could be construed as a potential conflict of interest.
